# Application of machine learning approaches to administrative claims data to predict clinical outcomes in medical and surgical patient populations

**DOI:** 10.1371/journal.pone.0252585

**Published:** 2021-06-03

**Authors:** Emily J. MacKay, Michael D. Stubna, Corey Chivers, Michael E. Draugelis, William J. Hanson, Nimesh D. Desai, Peter W. Groeneveld

**Affiliations:** 1 Department of Anesthesiology and Critical Care, Perelman School of Medicine at the University of Pennsylvania, Philadelphia, Pennsylvania, United States of America; 2 Penn Center for Perioperative Outcomes Research and Transformation (CPORT), University of Pennsylvania, Philadelphia, Pennsylvania, United States of America; 3 Penn’s Cardiovascular Outcomes, Quality and Evaluative Research Center (CAVOQER), University of Pennsylvania, Philadelphia, Pennsylvania, United States of America; 4 Penn Predictive Healthcare, Penn Medicine, University of Pennsylvania, Philadelphia, Pennsylvania, United States of America; 5 Division of Cardiovascular Surgery, Perelman School of Medicine at the University of Pennsylvania, Philadelphia, Pennsylvania, United States of America; 6 Leonard Davis Institute of Health Economics (LDI), University of Pennsylvania, Philadelphia, Pennsylvania, United States of America; 7 Department of Medicine, Perelman School of Medicine at the University of Pennsylvania, Philadelphia, Pennsylvania, United States of America; 8 Corporal Michael J. Crescenz Veterans Affairs Medical Center, Philadelphia, Pennsylvania, United States of America; Vellore Institute of Technology: VIT University, INDIA

## Abstract

**Objective:**

This study aimed to develop and validate a claims-based, machine learning algorithm to predict clinical outcomes across both medical and surgical patient populations.

**Methods:**

This retrospective, observational cohort study, used a random 5% sample of 770,777 fee-for-service Medicare beneficiaries with an inpatient hospitalization between 2009–2011. The machine learning algorithms tested included: support vector machine, random forest, multilayer perceptron, extreme gradient boosted tree, and logistic regression. The extreme gradient boosted tree algorithm outperformed the alternatives and was the machine learning method used for the final risk model. Primary outcome was 30-day mortality. Secondary outcomes were: rehospitalization, and any of 23 adverse clinical events occurring within 30 days of the index admission date.

**Results:**

The machine learning algorithm performance was evaluated by both the area under the receiver operating curve (AUROC) and Brier Score. The risk model demonstrated high performance for prediction of: 30-day mortality (AUROC = 0.88; Brier Score = 0.06), and 17 of the 23 adverse events (AUROC range: 0.80–0.86; Brier Score range: 0.01–0.05). The risk model demonstrated moderate performance for prediction of: rehospitalization within 30 days (AUROC = 0.73; Brier Score: = 0.07) and six of the 23 adverse events (AUROC range: 0.74–0.79; Brier Score range: 0.01–0.02). The machine learning risk model performed comparably on a second, independent validation dataset, confirming that the risk model was not overfit.

**Conclusions and relevance:**

We have developed and validated a robust, claims-based, machine learning risk model that is applicable to both medical and surgical patient populations and demonstrates comparable predictive accuracy to existing risk models.

## Introduction

Accurately estimating risk is critical for decision making in both surgical and medical patient populations [1]. But existing risk models are limited by an inability to rapidly obtain accurate information regarding patient risk [2–4], only apply to certain subsets of patient populations [2–8], and become outdated quickly because these models are not build to be continuously updated with new data [2–8]. For instance, while logistic regression risk models exist for both medical [5–8] and surgical populations [2–4], these risk models require time-consuming, manual data entry [2–4], only apply to limited subsets of patients (e.g. undergoing one type of surgical procedure [2–4] or with a specific medical diagnosis [5–8], and become outdated because such models cannot keep up with the continual improvements in healthcare treatments [2–8].

With advancements in computational power, paired with exponential increases in available data, artificial intelligence (AI) and machine learning (ML) have become indispensable tools in the technology industry [9,10]. In healthcare, ML and deep-learning techniques have already contributed to important advances in medical imaging diagnostics in the fields of radiology [11,12], neuroradiology [13], and dermatology [14,15]. Outside of medical imaging, robust ML algorithms for prognostication have been developed primarily using electronic medical record (EMR) data [16–18]. However, such models’ reliance on EMR data, which is often proprietary and unique to a particular health system, makes implementing these prognostic tools across multiple health systems costly and challenging.

Claims data represent an underutilized data source to develop a ML prognostic model that is not only robust and accurate–but scalable. While admittedly, claims data are less granular than EMR data, we hypothesized that a ML algorithm developed using claims data would offer predictive accuracy comparable to, or exceeding currently used risk models. The primary goal of this study was to develop and validate a claims data-based, ML prognostic model that would address the current limitations of existing risk models such as manual data entry, lack of generalizability, and quickly outdated risk estimates [2–8]. We hypothesized that a ML prognostic risk model using claims data would demonstrate accuracy comparable to or exceeding that of existing risk models, provide a platform for automatic data entry, offer immediate risk estimates to clinicians, and develop this ML model with software to allow for continuous updates as new data becomes available. Thus, we therefore developed and validated a claims-based ML prognostic model that provides comprehensive risk estimates of mortality, rehospitalization, and 23 clinical adverse events among both surgical and medical inpatients. Built as the University of Pennsylvania’s Stage 1 submission for the Centers for Medicare and Medicaid Services (CMS) AI Health Outcomes Challenge [19], our ML risk model is a prototype; ultimately designed to leverage CMS’s entire inpatient population for maximum applicability. Moreover, we developed a fully automatable, individualized risk calculator software tool designed to present the output of this ML prognostic model in an intuitive, easily interpretable format for use by healthcare providers.

## Materials and methods

### Data source

This study used a limited data set (LDS) of a random 5% sample Medicare beneficiaries encompassing fee-for-service (FFS) claims data–Parts A/B claims from 2008–2011. These data were linked to the publicly available US Census data by county code. LDS data was obtained following the completion of a CMS Data Use Agreement (DUA) for both the training, and the test datasets. These data were obtained in accordance terms of participation in Stage 1 of the CMS Artificial Intelligence (AI) Health Outcomes Challenge [20]. This project was deemed exempt by the University of Pennsylvania IRB and informed consent was waived.

### Study population

Inclusion criteria consisted of any fee-for-service, Medicare beneficiary aged 18 years or older with an inpatient hospitalization (admitted for either medical care or a surgical procedure), between January 1, 2009 and December 31, 2011. Inpatient claims from the year prior to the randomly-selected, inpatient, index admission were used to indicate preexisting conditions for a given beneficiary. Detailed information on data cleaning and cohort development may be found in the **eSupplement** (S1 File).

### Model input: Covariates

We used industry-standard categorizations applied to the raw Medicare Part A and Part B data elements of each hospitalization to generate a set of categorized features. Comorbidity classification used the existing hierarchical condition categories (HCC) [8,21], diagnosis related groupings (DRG) [22], and surgical and procedural classification using the clinical classification software (CCS) [23] categorizations; purposefully chosen because developed a risk model applicable to both medical and surgical patient populations. *ICD-9-CM* diagnoses codes were mapped to HCCs. Both *ICD-9-CM* procedure codes and current procedural terminology (CPT) physician billing codes were mapped to CCS categories. Detailed information on data categorization–including exact variables used for grouping into HCC, CCS, and DRG categories–may be found in the **eSupplement** (S2 File).

Broadly speaking, our ML risk model features originate from the claims data columns that describe: (a) Beneficiary demographic data, (e.g. age, sex, and race); (b) geographic data (e.g. state and county); (c) Medicare indicator codes (e.g. End-stage renal disease (ESRD) indicator, Medicare status indicator (disability, ESRD, or neither)); (d) prior and present on admission claim ICD-9 diagnoses codes categorized into HCC categories; (e) prior claim procedure codes (CPT and/or ICD-9 procedure codes) categorized into CCS categories; (f) DRG code for the index inpatient admission; (e) hospitalization data at the time of admission (e.g. admission source (home, ER, transfer, etc.), (f) US census data (e.g. median income, percent unemployed, percent below poverty, household size, percent married, percent with high school education, percent with bachelor’s degree, percent car commute to work; (g) hospitalization data at the time of discharge (e.g. length of stay and discharge status) was used for the rehospitalization model, calculated at the time of discharge. Please refer to **eSupplement** (S3 File) for detailed information on how US Census Data was incorporated into the ML risk model.

HCCs were used to indicate both preexisting comorbidities and subsequent complications (e.g. ‘adverse events’). To differentiate between comorbidities and ‘adverse events,’ we combined two techniques. One, an HCC was classified as preexisting if the *ICD-9-CM* diagnosis codes (mapped to HCCs) appeared in any inpatient, outpatient, home health, skilled nursing facility, or physician billing claims starting January 1, 2008 and extending up to the admission date for the index admission. That is–an *ICD-9-CM* code from a claim with a date previous to the index admission date was used to indicate preexisting comorbid disease. Two, an HCC was classified as preexisting if the *ICD-9-CM* diagnosis code(s) had a corresponding “present on admission” (POA) indicator variable from the index admission. Detailed information the development of model features and heuristics may be found in the **eSupplement** (S4 File).

### Model output: Outcomes

Our ML risk model was designed to be used as a clinical decision-support tool at the time of patient admission or discharge, and provide a broad suite of estimates of clinically important patient-specific health outcomes. The primary outcome of mortality at 30-days was defined using death information obtained from the Social Security Administration’s Death Master File–shown to be a reliable indicator of mortality for Medicare beneficiaries [24].

The secondary outcome of rehospitalization within 30-days was defined if a given beneficiary was readmitted to a hospital for a subsequent inpatient hospitalization within 30-days of discharge from an inpatient hospitalization. The logic used to define rehospitalization, along with a corresponding diagram may be found in the **eSupplement**, (S4 File; **eFig 1**). Additional secondary, adverse event outcomes were defined by *new* HCC categories (i.e. not a preexisting HCC) during the index admission or within the 30-day timeframe. The adverse events were chosen based on a combination of changes in frequency of occurrence and clinical expertise. Details on the process for determining which HCCs were chosen as adverse event outcomes may be found in the **eSupplement** (S4 File).

### Machine learning algorithms

For maximum flexibility in using our ML models in data visualization presentations and interpreting their predictions, we choose to develop a separate model for each model output. That is, we developed individual models to estimate the likelihood of mortality at 30 days, the likelihood of rehospitalization within 30 days, and the likelihood of each of the 23 individual adverse events at 30 days, for a total of 25 independent risk models.

We employed best-practices [25] for objectively assessing model performance and robustness while guarding against overfitting. Specifically, we used 75% of the data for training a candidate model and the remaining 25% of the data to test and verify the accuracy and robustness of the model. We fit models of the following types: (1) logistic regression; (2) support vector machine; (3) random forest; (4) multi-layer perceptron neural net; (5) two variations of gradient boosted trees. In addition, we fit aggregate models comprised of combinations of two or more different types of models aggregated by consensus voting.

### Data preparation and feature engineering

All model development and evaluation work was performed using Python v3.7.6 [26]. Notable open-source Python packages used were: NumPy [27], SciPy [28], Pandas [29], SciKit-Learn [30–32], and XGBoost [33]. Detailed information on data preparation, coding, variable weighting methods, summaries of how each variable was transformed, and the resulting number of variables generated may be found in the **eSupplement**, (S5 File).

### Model performance assessment and statistical analysis

Model hyperparameters were tuned on the training data set using cross validation and final model predictions were made on the test data set (S5 File). Model performance was assessed using two metrics: (1) the area under the receiver operator curve (AUROC) and (2) the Brier Score. The AUROC is the sensitivity vs 1 minus the specificity plot, and should not be used in isolation as a performance metric because it is only a measure of discrimination, not calibration [4,34]. The Brier score is the average squared difference between the predicted probability and the observed outcome, and reflects both discrimination and calibration [35,36]. As a given model’s AUROC approaches zero for nonevents and one for events, the Brier score will decrease–approaching zero being optimal with an upper limit of acceptability equal to 0.25 [36]. Model performance was also evaluated by calculating the positive predictive value (PPV) at varying thresholds–termed “alert rates” (AR) (e.g. the PPV at an AR of 1%, 2%, 10%, etc.)–along with a data visualization plotting the alert rate against the true positive rate. A 95% confidence interval (CI) for each AUROC was calculated using bootstrapping [37]. Details on model performance assessment and statistical analysis is presented in **eSupplement** (S5 File).

### Independent dataset testing and model performance assessment

An independent, test dataset was used to evaluate our ML risk model. This test dataset contained a second, random 5% sample of fee-for-service Medicare beneficiaries from the subsequent calendar year; January 1, 2012 –December 31, 2012. Our model was retrained using 100% of the original dataset (January 1, 2009 –December 31, 2011) and retested on the new, independent, test dataset. Model performance was assessed using the same parameters as the original model evaluation–AUROC and Brier Score.

### Development: Clinician-facing risk software tool

Once all 25 models were fitted, these were then used to power individualized patient-specific risk visualizations that summarize our ML Model’s output risk estimates for any patient for any time period. For each outcome of interest, the Extreme Gradient Boosted (XGB) model was used for quantifying a risk score. Then, the corresponding logistic regression (LR) model was used for computing easily interpretable “contributing factors” to that risk score.

The individualized patient-specific data visualizations were generated as follows:

1. Patient risk estimates were generated for mortality, unplanned admission, and their individual, most likely adverse events using the XGB model and the patient’s specific input characteristics.
2. Population distributions of risk and calculated using the XGB model across all patients undergoing the same procedure as this patient, or in the absence of a specific procedure, all patients close in age to this patient.
3. Contributing factors were generated for mortality, unplanned admission, and most likely adverse events using the LR model evaluated for this patient. Because the LR model consists of a simple linear combination of features, feature sizes are thus estimated and can be displayed as “contributing factors” comprising a final risk score.
4. Clinically-determined risk criteria were defined to determine when to display message boxes with below average risk, above average risk, and relatively elevated risk messages.

## Results

### Study population

From the 2,792,785 Medicare beneficiaries there were 770,777 beneficiaries that met the inclusion criteria of an inpatient admission between January 1, 2009 and December 31, 2011, and used in the development of the ML risk model. This dataset of 770,777 beneficiaries was then split into a training (578,083, 75%) and a test (192,694, 25%) dataset.

### Model prediction and performance results

The overall event rate was 8.7% for 30-day mortality, 9.1% for rehospitalization, 30-day adverse event rates ranged from 0.23% for opportunistic infections to 6.0% for cardio-respiratory failure and shock. The AUROC was 0.88 for 30-day mortality, 0.73 for rehospitalization at 30-days, and ranged from 0.74 for drug and alcohol psychosis to 0.87 for respiratory dependence and tracheostomy status (Table 1). The Brier scores were low across all outcomes–approaching zero being optimal. The Brier score was 0.061 for 30-day mortality, 0.076 for rehospitalization at 30-days, and ranged from 0.002 for both acute diabetes complication and opportunistic infection to 0.051 for cardio-respiratory failure or shock (Table 1).

**Table 1:** Results of prediction models.

Target Events	Base Frequency (%)	AUROC	95% CI for AUROC	Brier Score
Mortality	8.74	0.88	[0.88, 0.88]	0.06
Rehospitalization (inpatient or SNF)	9.10	0.73	[0.73, 0.74]	0.07
Acute renal failure (HCC 135)	3.88	0.79	[0.79, 0.79]	0.04
Artificial openings for feeding/elimination (HCC 188)	1.18	0.85	[0.84, 0.86]	0.01
Cardio-respiratory failure/shock (HCC 84)	6.01	0.80	[0.80, 0.81]	0.05
Coma/brain compression/anoxia (HCC 80)	0.85	0.80	[0.79, 0.81]	0.01
Diabetes/acute complication (HCC 17)	0.27	0.86	[0.84, 0.87]	<0.01
Drug/alcohol psychosis (HCC 54)	0.74	0.74	[0.72, 0.75]	0.01
Head trauma/brain bleed: (HCCs 166, 99, 167)	0.87	0.78	[0.77, 0.80]	0.01
Hemiplegia/hemiparesis (HCC 103)	1.46	0.86	[0.86, 0.87]	0.01
Hip fracture/dislocation (HCC 170)	0.62	0.86	[0.85, 0.87]	0.01
Implanted device/graft complication (HCC 176)	1.24	0.76	[0.75, 0.77]	0.01
Intestinal obstruction/perforation (HCC 33)	2.42	0.80	[0.80, 0.81]	0.02
Ischemic/unspecified stroke (HCC 100)	3.26	0.83	[0.83, 0.84]	0.03
Limb amputation: (HCCs 173, 189)	0.71	0.82	[0.80, 0.83]	0.01
Lung injury/pneumonia (HCCs 115, 114)	2.67	0.81	[0.80, 0.82]	0.03
Monoplegia/other paralytic (HCC 104)	0.34	0.82	[0.80, 0.84]	<0.01
Myocardial ischemia/infarction: (HCCs 86, 87)	3.00	0.80	[0.80, 0.81]	0.03
Opportunistic infections (HCC 6)	0.26	0.76	[0.74, 0.78]	<0.01
Protein-calorie malnutrition (HCC 21)	2.20	0.78	[0.77, 0.79]	0.02
Respiratory arrest (HCC 83)	0.47	0.82	[0.81, 0.84]	0.01
Respiratory dependence/tracheostomy status (HCC 82)	0.87	0.87	[0.86, 0.88]	0.01
Sepsis (HCC 2)	3.48	0.81	[0.80, 0.81]	0.03
Severe infection: skin/muscle/tendon/bone: (HCCs 162, 154, 157, 39, 106)	0.77	0.81	[0.80, 0.83]	0.01
Spinal cord injury/quadriplegia: (HCCs 72, 70)	0.80	0.80	[0.78, 0.81]	0.01

Abbreviations: **AUROC**: Area under the receiver operating curve; **CI**: Confidence interval; **SNF**: Skilled nursing facility; **HCC**: Hierarchical condition categories.

### Model evaluation assessment results

**Fig 1:**
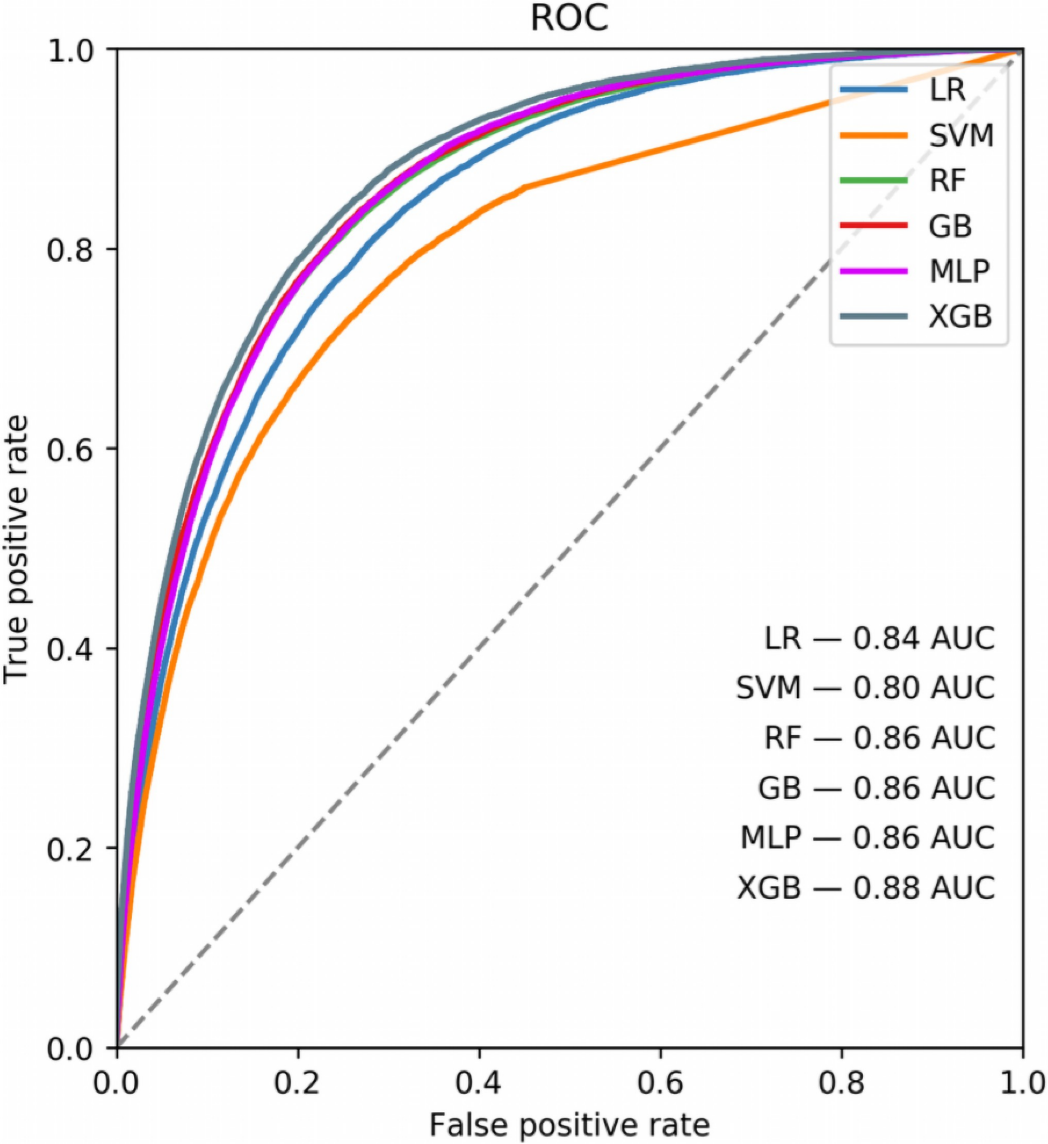
Algorithm performance comparisons for predicting 30-day mortality. Model area under the curve (AUC) comparisons for 30-day mortality. Abbreviations: Logistic regression (LR), supported vector machine (SVM), random forest (RF), multilayer perceptron (MLP), extreme gradient boosted tree (XGB).

Model hyperparameters were tuned on the training data set using cross validation and final model predictions were made on the test data set. Fig 1 shows a summary of the AUROC performance metric for different model types fit to the same data set predicting the 30-day mortality outcome. As can be seen in Fig 1, the AUROC metric is quite close among all models, with extreme gradient boosted tree (XGB) performing slightly better than the other models.

**Fig 2:**
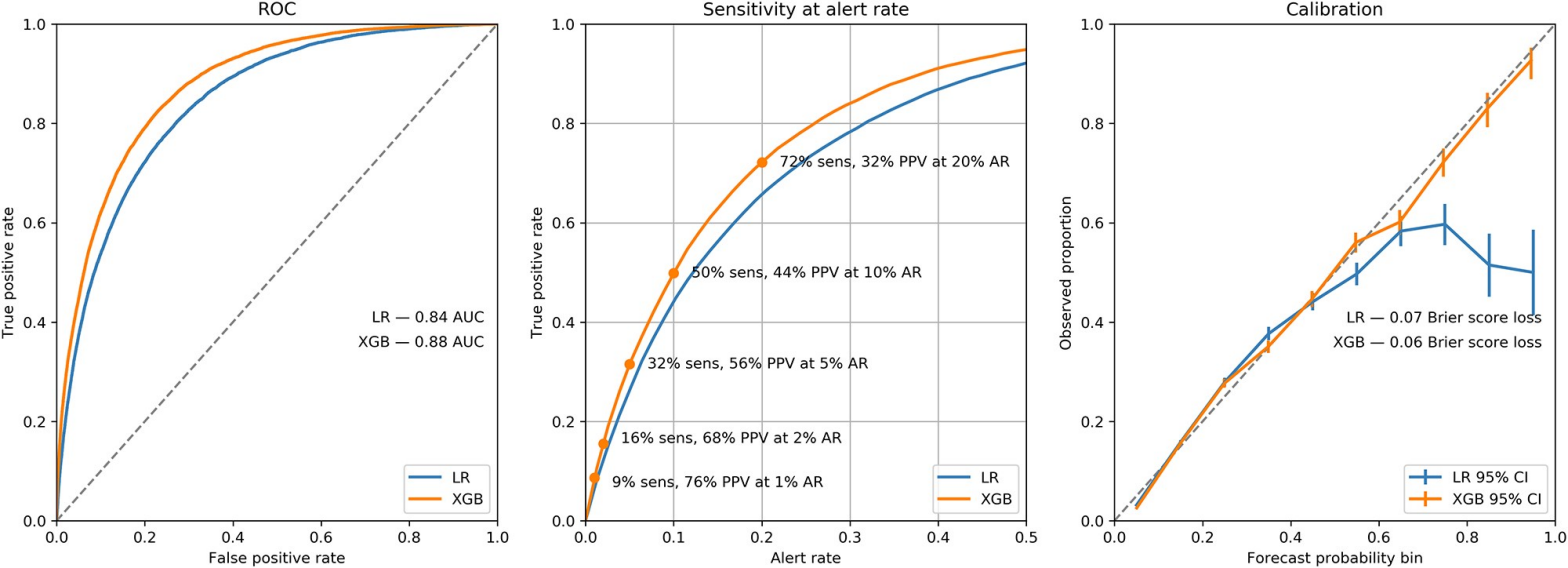
Test data algorithm performance comparisons for predicting 30-day mortality. The left panel displays the full area under the curve (AUC) for logistic regression (LR) algorithm vs extreme gradient boosted (XGB) algorithm. The middle panel displays the true positive rate vs the alert rate (e.g. 1% alert rate would capture the 1% at highest risk for 30-day mortality) at selected alert rates (1%, 2%, 5%, 10%, and 20%), along with details about the sensitivity and positive predictive value (precision) at those alert rates for the LR and XGB models. The right panel of displays calibration curves along with the Brier score loss–an indication of the overall calibration of the LR and XGB models.

**Fig 3:**
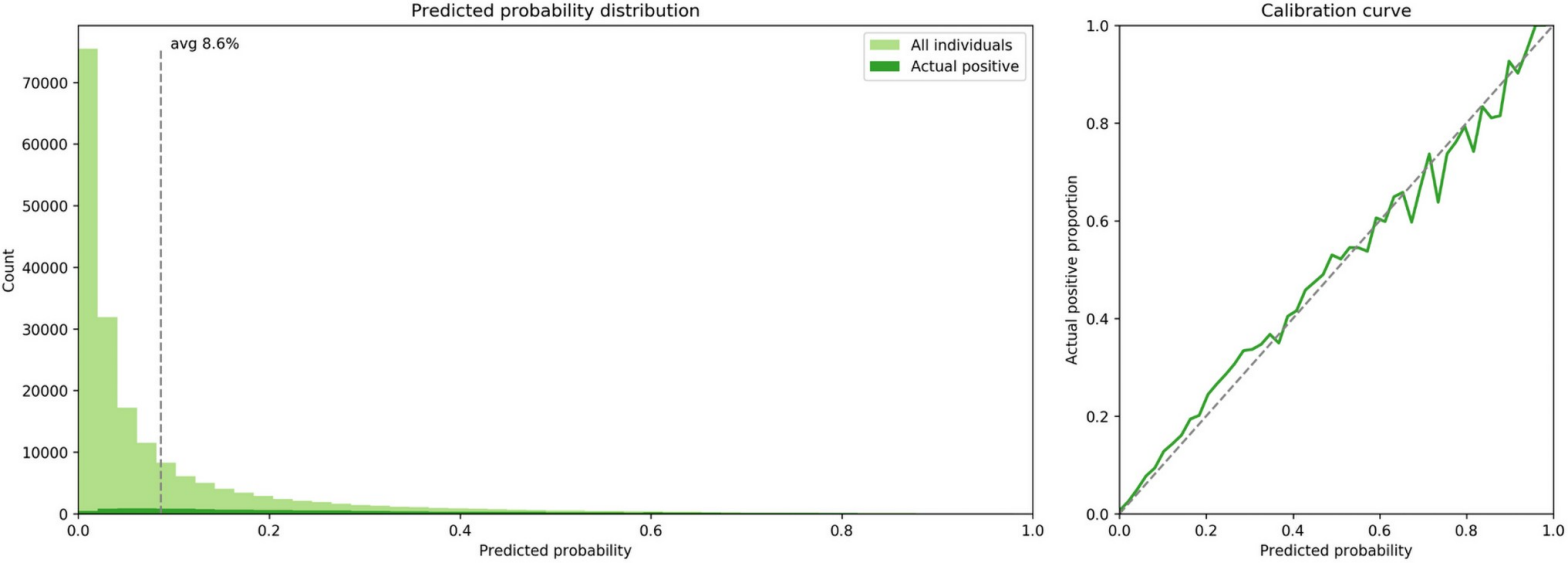
The Extreme gradient boosted tree model calibration for predicting 30-day mortality. The **left pane**l shows that the extreme gradient boosted (XGB) tree model produces a wide range of risk estimates for the test data set individuals, from 0.0 to 1.0. The **right pane**l is a calibration curve and shows that each risk score bin accurately represents the true average mortality rate of those individuals in that bin. In other words, the right panel shows that the predicted mortality curve (green line) follows the actual mortality curve (dashed line) closely.

**More** detailed metrics on performance assessment for these two models–XGB and LR–on the 30-day mortality test data set can be seen in Fig 2. Additionally, Fig 3 is a data visualization of the predicted mortality by the XGB model compared to the actual, 30-day mortality in the test dataset. The same data visualizations for model performance assessment of rehospitalization and one example of an adverse event (cardiorespiratory failure/shock) may be found in the **eSupplement** (S5 File; **eFigs 5–8**). All adverse event models demonstrated similar performance results (as presented in Table 1). A median adverse event data visualization for all 23 adverse events may be found in the **eSupplement: (**S5 File; **eFig 9)**.

### Independent dataset testing: Model performance assessment results

From the 2,674,730 Medicare beneficiaries in the second, independent, test dataset, there were 332,653 beneficiaries that had at least one inpatient hospitalization within the time periods specified by CMS for model testing. Because there was no calendar overlap between the, second, test dataset from 2012 with the original dataset from 2009–2011, we retrained our ML risk model using the original dataset in its entirety (770,777 beneficiaries) and retested on the independent, test data sample (332,653 beneficiaries). Model performance assessment results were consistent with the results from the original ML risk model as presented above; indicating our model was not overfit. Results of model testing on this independent dataset may be found in the **eSupplement** (S6 File).

### Clinician-facing software tool development

**Fig 4:**
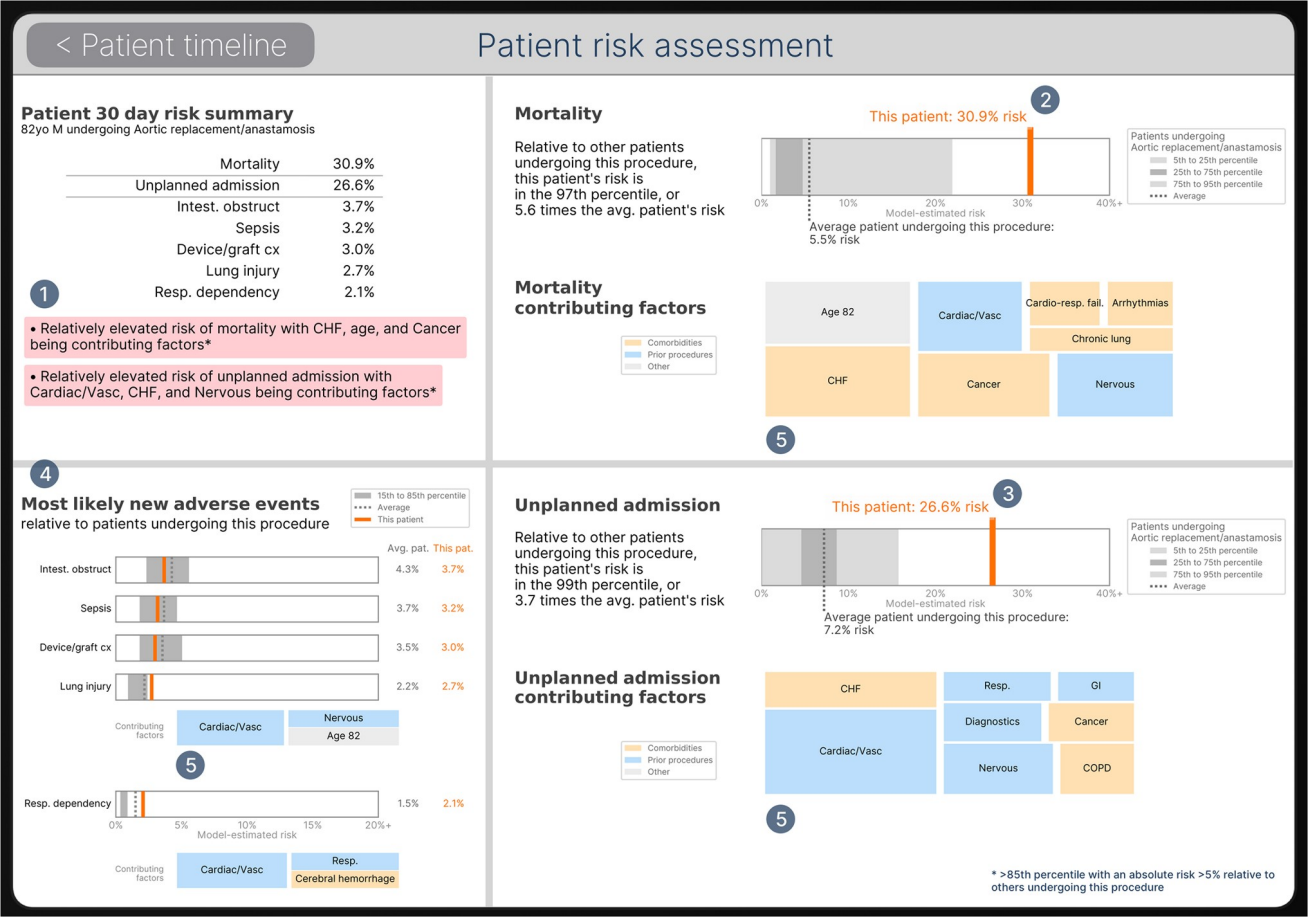
Example output from clinician-facing software risk tool. Descriptors on risk context and contributors correspond to the numbers in **Fig 4** as follows: (1) Clinically-determined risk criteria were defined to determine when to display message boxes with below average risk (green), above average risk (yellow), and relatively elevated risk (red) messages. (2) Patient’s individual risk of 30-day mortality (as estimated by the XGB model) is presented as relative to the average risk of 30-day mortality among all patients undergoing the same procedure (e.g. aortic surgery). (3) Patient’s individual risk of unplanned admission is presented as relative to the average risk of unplanned admission among all patients undergoing the same procedure (e.g. aortic surgery). (4) Summarizes individualized risk of the most likely adverse events; presented as relative to the average risk for a given adverse event among all patients undergoing the same procedure (e.g. aortic surgery). (5) Factors (e.g. preexisting comorbid diseases, or previous procedures) contributing to risk are scaled and sized (as estimated by logistic regression) to reflect relative contribution to overall risk.

Once all 25 ML risk models were fitted, they were then used to power individualized patient-specific risk visualizations that summarize the model output risk estimates for any patient at 30 days. For each outcome of interest, the XGB model was used for quantifying a risk score and the corresponding LR model was used for computing easily interpretable “contributing factors” to that risk score. Fig 4 shows an example patient risk visualization at 30-days for a patient undergoing open aortic surgery.

## Discussion

We developed an accurate, robust, and interpretable risk model using CMS claims data that produces 30-day estimates of mortality, rehospitalization, and 23 adverse events, including the predicted probability of each outcome’s occurrence. Our claims-based XGB model achieved very high predictive accuracy for 30-day mortality and adverse events, with an AUROC of 0.88 for 30-day mortality, and an AUROC range of 0.74–0.87 for other adverse clinical events. The model’s predictive accuracy for rehospitalization was more modest, with an AUROC of 0.73. To provide additional clinical interpretability, we augmented prediction results as calculated from the XGB model with contributing risk factors calculated using a supplemental logistic regression model.

The AUROC range of 0.74–0.87 for our ML risk model is comparable to existing surgical [2,4], and medical [5–8] risk models. For surgical patient populations, AUROC estimates for mortality and adverse events ranges from 0.62 to 0.83 for the Society of Thoracic Surgeons (STS) risk model [2], and from 0.80 to 0.94 for the American College of Surgeons (ACS) National Surgical Quality Improvement Program (NSQIP) risk model [4]. For medical patient populations, recent claims-based AUROC risk estimates for mortality range from 0.69 to 0.83 [5]. For rehospitalization, our model’s performance was comparable to the AUROC of existing rehospitalization prediction models such as: LACE index [38], LACE+ index [39], and HOSPITAL score [40]; with AUROC estimates ranging from 0.69 to 0.77 [38–40].

Current risk prediction models have not achieved widespread adoption, possibly related to limitations in generalizability, lack of automation, and inability to update predictions based on new data [2,4,38–40]. The registry-based STS [3] and the NSQIP [4] risk models only apply to surgical patient populations [3,4], and the claims-based prognostic models only apply to certain subsets of medical patients [5–8]. But even risk models such as STS and NSQIP are often underutilized, and lack of automation may be the culprit. Several existing prognostic models require cumbersome, manual data entry [2,4–8,38–40]; a significant barrier to use in the busy clinical setting. Moreover, predictions derived from static prediction models quickly become outdated because without automated data”refreshing” of these models, improvements in clinical outcomes secondary to advancements in clinical therapies cannot be captured in an expeditious manner. Moreover, because of proprietary differences in electronic medical record (EMR) systems across the United States, EMR-based ML prognostic risk models have limited generalizability.

Our claims-based, ML risk model and software tool have addressed limitations of existing risk models in three important respects. First, our ML risk model and software tool was designed to be fully automatable; which may facilitate increased use in the busy clinical setting. Second, our claims-based ML was developed to operate outside of any single, proprietary EMR; potentially increasing the generalizability with use across a variety of different types of health systems. Third, our automated, claims-based ML model was developed to be able to be updated by incorporating new data as it becomes available. This approach would facilitate continuous refining, testing, and improvement of our ML risk model’s predictions. The strength of our integrated ML model and software tool does not solely reside in its prognostic ability, but also with its potential for widespread deployment, pragmatic use, and model enhancements over time.

## Limitations

It is important to acknowledge several limitations to this study. First, our model was developed on a relatively small fraction of CMS inpatient claims (made available to us through the CMS AI Health Outcomes Challenge) [20]; representing only 2% of Medicare fee-for-service hospitalizations in 2008–2011). While these were a random sample of hospitalizations, it is likely that development using a larger, and more recent, set of claims data would improve predictive accuracy. Second, while we did incorporate outpatient claims data from a given beneficiary, we did not have the availability of prescription claims, which could have improved outcomes predictions. Third, while we did compare the multilayer perceptron model in our analysis, it did not outperform our tree-based models and due to time constraints related to the Challenge [20], we didn’t attempt more complex, deep learning techniques, such as deep neural network models [41,42], which may have improved the accuracy and precision of our ML model. Fourth, our ML risk model was developed solely from CMS Medicare data; limiting the model’s generalizability to patients covered by Medicaid or by private payer insurance. Fifth, while we believe our risk calculator software tool potentially has high usability, it has not been tested by healthcare providers in the real-world setting. Prototype testing with both healthcare providers and patients will be an important next step in developing a pragmatic software tool for risk.

## Conclusions

We have developed and validated an accurate, robust, and interpretable ML risk model using CMS claims data among a large, Medicare patient population. Our claims-based ML risk model demonstrated comparable predictive accuracy to currently used medical and surgical predictive models, and may provide a platform for future automation for ease of use as a clinical decision support tool.

## Supporting information

S1 File Step 1 of model development—Data cleaning & cohort development. (PDF)

S2 File Step 2 of model development—Data categorization. (PDF)

S3 File Step 3 of model development—Supplemental data sources. (PDF)

S4 File Step 4 of model development—Constructed features. (PDF)

S5 File Step 5 of model development—Evaluation of strengths & weaknesses. (PDF)

S6 File Model performance on independent, test dataset. (PDF)
