# Supplementary material for: Application of machine learning approaches to administrative claims data to predict clinical outcomes in medical and surgical patient populations
Source: PLoS One. 2021 Jun 3;16(6):e0252585. doi: 10.1371/journal.pone.0252585 (PMC8174683; doi:10.1371/journal.pone.0252585)
Supplement: S1 File — (PDF) [file pone.0252585.s001.pdf]

Supplement 1: Step 1 of Model Development – Data Cleaning & Cohort Development

S1 Text: Step 1: Data Cleaning & Cohort development

We performed steps necessary to clean the data and develop the cohort. The following will describe that process in detail, and reference the relevant coding scripts.

Step 1.a: Data Preparation and Cleaning

First, we manually reviewed all data files and data columns to determine data inputs for the model features. A key was created that listed all the data columns, the data column row, the data column label, and differences in data column order and label(s) between the 2008 – 2010 files vs the 2011 files were indicated.

Second, we created data key files that indicated which data columns should be included from each data file and read into separate .csv files. This process had to be performed separately for (a) the 2008 – 2010 files; (b) 2011 data files; and (c) supplemental 2009 date files. Once data columns and data files had been identified, these data were read in (for each file and corresponding year). It should be noted that column headers had to be added to all files. First, headers were added to the 2008 – 2010 files. Second, the 2011 data columns differed from the 2008 – 2010 data columns two important ways. One, data columns were ordered (e.g. sorted) differently. Two, data columns were labeled with different column headers (e.g. labels). Therefore, the 2011 data files were sorted and relabeled to correspond to the sort order and header labels that matched the 2008 – 2009 data files.

Third, we combined the filtered dataset into one set of files across all years. In order to accomplish this successfully several datasets had to be handled differently: (1) carrier claims files from 2011 and 2010 and (2) the supplemental data file from 2009. First, we will address the carrier claims data. The 2011 carrier claims data were split into two files; one carrier claim file and one carrier line file, each containing different data columns. The 2010 carrier claims data were also split into two files, but not in the same way the 2011 carrier claims data were split. The 2010 carrier claims data were split into two sequential files containing the same data. Different from both 2010 and 2011, all the carrier claims data from 2008 – 2009 were contained under one carrier claim file per year: 2008 and 2009. The 2011 carrier claims and line files were unified, relabeled to correspond to the 2008 – 2010 carrier claims files, and the 2010 carrier claims files (a and b) were unified. Second, the supplemental date file for 2009 had to be joined to each of the 2009 files selected for inclusion in the model. Once these differences in the data had been addressed, files from a single year were merged across all types of claims.

Finally, the compiled dataset from each year was appended sequentially – resulting in a single data file encompassing all claims from all years.

Step 1.b: Cohort Development

Model inclusion criteria for cohort development consists of: any beneficiary with at least one inpatient hospitalization between January 1, 2009 and December 31, 2011.

Our beneficiary selection script includes any beneficiary with an inpatient claim admit date (corresponding to the 'CLM_ADMSN_DT' data column) between January 1, 2009 and December 31, 2011 from any of the inpatient claim files. See eTable 1 for detailed info. Note that while 2008 inpatient claims are not used in the beneficiary selection script itself because the claim dates are limited to quarterly bucket resolution, they are however used when calculating model features for the selected beneficiaries.

eTable 1

Year	Admit variable label	Inpatient claims file[a]
2008[b]	N/A	N/A
2009	CLM_ADMSN_DT	Part A claims
2010	CLM_ADMSN_DT	Part A claims
2011	CLM_ADMSN_DT	Part A claims
[a] Note: the inpatient files are used to define the beneficiary inclusion criteria. These do not represent all the files used to create the prediction model<br>[b] Note: the 2008 data files did not include days, and so could be used in the beneficiary selection process. However, data files from this year are used later when calculating model features.		
