# Supplementary material for: Application of machine learning approaches to administrative claims data to predict clinical outcomes in medical and surgical patient populations
Source: PLoS One. 2021 Jun 3;16(6):e0252585. doi: 10.1371/journal.pone.0252585 (PMC8174683; doi:10.1371/journal.pone.0252585)
Supplement: S2 File — (PDF) [file pone.0252585.s002.pdf]

Supplement 2: Step 2 of Model Development – Data Categorization

S2 Text: Step 2: Data Categorizations

We used industry-standard categorizations applied to the raw Medicare Part A and Part B data elements of each hospitalization to generate a set of categorized features. Comorbidity classification used the existing hierarchical condition categories (HCC),(1, 2) diagnosis related groupings (DRG),(3) and surgical and procedural classification using the clinical classification software (CCS)[(4)] categorizations; purposefully chosen because developed a risk model applicable to both medical and surgical patient populations. Icd-9-cm diagnoses codes were mapped to HCCs. Both icd-9-cm procedure codes and current procedural terminology (CPT) physician billing codes were mapped to CCS categories. Generation of these categorizations through the use of clinically-relevant groupings ensure that the final AI model will be able to be used in the clinician-facing Individualized Risk Calculator tool either in the context of or without access to specific Medicare billing codes. Details on how the HCCs, DRGs, and CCS categorizations were employed as features in the model will be described in detail in Step 4. eTable 2 indicates which data columns (from all the applicable claim files) were mapped into HCCs, CCS, and DRG categorizations.

eTable 2

Constructed feature	Data columns (2008-2010) [a]	Data columns (2011) [a] (if different than 2008-2010 – renamed to the corresponding 2008-2010 variable)
HCC Categories	CLM_DGNS_CD1-10<br>CLM_PRNCPAL_DGNS_CD<br>CLM_ADMTG_DGNS_CD<br>CLM_POA_IND_SW1-10	ICD_DGNS_CD1-10<br>PRNCPAL_DGNS_CD<br>ADMTG_DGNS_CD<br>CLM_POA_IND_SW1-10
CCS Categories (icd-9 codes)	CLM_PRCDR_CD1-6<br>CLM_PRCDR_PRFRM_DT1-6	ICD_PRCDR_CD1-6<br>PRCDR_DT1-6
CCS Category (CPT codes)	LINE_HCPCS_CD1-13	HCPCS_CD
DRG Category	CLM_DRG_CD	CLM_DRG_CD
[a]Note: the inpatient files are used to define the beneficiary inclusion criteria. These do not represent all the files used to create the prediction model		

S2 Supplement References

1. Pope GC, Kautter J, Ellis RP, Ash AS, Ayanian JZ, Lezzoni LI, et al. Risk adjustment of Medicare capitation payments using the CMS-HCC model. Health Care Financ Rev. 2004;25(4):119-41.
2. CMS.gov. Centers for Medicare & Medicaid Services [Available from: https://www.cms.gov
3. (CMS) CfMMS. ICD-10-CM/PCS MS-DRG v34.0 Definitions Manual [Available from: https://www.cms.gov/ICD10Manual/version34-fullcode-cms/fullcode_cms/P0002.html.
4. (AHRQ) HCaUPHaAfHRaQ. Clinical Classification Software (CCS) for ICD-9-CM Fact Sheet [Available from: https://www.hcup-us.ahrq.gov/toolssoftware/ccs/ccsfactsheet.jsp.
