# Supplementary material for: Application of machine learning approaches to administrative claims data to predict clinical outcomes in medical and surgical patient populations
Source: PLoS One. 2021 Jun 3;16(6):e0252585. doi: 10.1371/journal.pone.0252585 (PMC8174683; doi:10.1371/journal.pone.0252585)
Supplement: S3 File — (PDF) [file pone.0252585.s003.pdf]

Supplement 3: Step 3 of Model Development – Supplemental Data Sources

S3 Text: Step 3: Supplemental Data Sources

We incorporated the publicly-available, linkable, US Census data.[(1)] While ideally we would have linked US Census data to beneficiary zip code, terms of the Limited Data Set (LDS) Data Use Agreement (DUA) prohibited the use of zip code data. Therefore, our Stage 1 submission incorporated census data at the county level, see eTable 3.

eTable 3

Census covariate constructed feature	Mapping file (icd9)	Data columns (2008-2010) [a]	Data columns (2011) [a] [If different than 2008-2010 – renamed to the corresponding 2008-2010 variable]
`Median income<br>`% unemployed<br>`% below poverty<br>`Household size<br>`% married<br>`% high school<br>`% bachelor<br>`% car commute to work	n/a	COUNTY_CODE	COUNTY_CODE
[a]Note: the inpatient files are used to define the beneficiary inclusion criteria. These do not represent all the files used to create the prediction model			

S3 Supplement References

1. Bureau USC. Open Data: Accessing Public Data. 2019.
