# Supplementary material for: Application of machine learning approaches to administrative claims data to predict clinical outcomes in medical and surgical patient populations
Source: PLoS One. 2021 Jun 3;16(6):e0252585. doi: 10.1371/journal.pone.0252585 (PMC8174683; doi:10.1371/journal.pone.0252585)
Supplement: S4 File — (PDF) [file pone.0252585.s004.pdf]

Supplement 4: Step 4 of Model Development – Constructed Features

S4 Text: Step 4: Constructed Features

Step 4.a: Model features and heuristics (Input)

Broadly speaking, the model features originate from the claims data columns that describe:

1. Beneficiary demographic data: age, sex, and race
2. Geographic data: state and county
3. Medicare indicator codes: ESRD indicator, Medicare status indicator (disability, ESRD, or neither)
4. Prior and present on admission claim ICD-9 diagnoses codes. These codes are further categorized into HCC categories. HCC diagnoses claims prior to the index admission are inputs for all prediction models. HCCs from the index admission,* not associated with a POA indicator are inputs for the unplanned admission model.
5. Prior claim procedure codes (CPT and/or ICD-9 procedure codes). These codes are further categorized into CCS categories. CCS procedure claims prior to the index admission are inputs for all prediction models. CCS procedures for index admission* are inputs for the unplanned admission model only.
6. DRG code for the index inpatient admission.
7. Hospitalization data at time of admission: admission source (home, ER, transfer, etc.), prior length of stay from previous inpatient admission(s)
8. County codes; These codes are linked to publicly available US census data covariates
    a. Median income
    b. Percent unemployed
    c. Percent below poverty
    d. Household size
    e. Percent married
    f. Percent high school
    g. Percent bachelors
    h. Percent car commute to work
9. Hospitalization at time of discharge: discharge destination, length of stay.

The full list of features used by the model, along with the source claim data columns are described in eTable 4.

eTable 4

Constructed feature	Data columns (2008-2010) [a]	Data columns (2011) [a] [if different than 2008-2010 – renamed to the corresponding 2008-2010 variable]
Beneficiary	DESY_SORT_KEY	DESY_SORT_KEY
State	STATE_CODE	STATE_CODE
County	COUNTY_CODE	COUNTY_CODE
Age	AGE<br>REFERENCE_YEAR	AGE<br>REFERENCE_YEAR
Race	RACE_CODE	RACE_CODE
Sex	SEX_CODE	SEX_CODE
Admission source code	CLM_SRC_IP_ADMSN_CD	CLM_SRC_IP_ADMSN_CD

Constructed feature	Data columns (2008-2010) [a]	Data columns (2011) [a] [if different than 2008-2010 – renamed to the corresponding 2008-2010 variable]
Prior inpatient visit count	CLM_ADMSN_DT	CLM_ADMSN_DT
Medicare Status Code	MEDICARE_STATUS_CODE	MEDICARE_STATUS_CODE
End-Stage Renal Indicator	ESRD_INDICATOR	ESRD_INDICATOR
HCC Categories*	CLM_DGNS_CD1-10<br>CLM_PRNCPAL_DGNS_CD<br>CLM_ADMTG_DGNS_CD<br>CLM_POA_IND_SW1-10	ICD_DGNS_CD1-10<br>PRNCPAL_DGNS_CD<br>ADMTG_DGNS_CD<br>CLM_POA_IND_SW1-10
CCS Categories (icd-9 codes)*	CLM_PRCDR_CD1-6<br>CLM_PRCDR_PRFRM_DT1-6	ICD_PRCDR_CD1-6<br>PRCDR_DT1-6
CCS Category (CPT codes)	LINE_HCPCS_CD1-13	HCPCS_CD
DRG Category	CLM_DRG_CD	CLM_DRG_CD
Census Covariates (as listed in eTable 3)	COUNTY_CODE	COUNTY_CODE
Length of stay [b]	Difference between:<br>CLM_ADMSN_DT and<br>NCH_BENE_DSCHRG_DT	Difference between:<br>CLM_ADMSN_DT and<br>NCH_BENE_DSCHRG_DT
Discharge status [b]	PTNT_DSCHRG_STUS_CD	PTNT_DSCHRG_STUS_CD
[a] Note: the inpatient files are used to define the beneficiary inclusion criteria. These do not represent all the files used to create the prediction model<br>[b] Indicates a covariate used only for rehospitalization ML risk model calculated at the time of discharge		

HCCs were used to indicate both preexisting comorbidities and subsequent complications (e.g. 'adverse events'). To differentiate between comorbidities and 'adverse events,' we combined two techniques. One, an HCC was classified as preexisting if the icd-9-cm diagnosis codes (mapped to HCCs) appeared in any inpatient, outpatient, home health, skilled nursing facility, or physician billing claims starting January 1, 2008 and extending up to the admission date for the index admission. That is – any icd-9-cm code from a claim with a date previous to the index admission date was used to indicate preexisting comorbid disease. Two, an HCC was classified as preexisting if the icd-9-cm diagnosis code(s) had a corresponding "present on admission" (POA) indicator variable. That is – any ICD-9-CM code from an index admission claim with an associated POA indicator was used to indicate preexisting comorbid disease and not an 'adverse event.'

Step 4.b: Model Predictions (Output)

Our approach was to create a clinical decision-support tool that can be used at the time of patient admission or discharge and provides a broad suite of estimates of clinically important patient-specific health outcomes. That is, the model will produce a comprehensive suite of 30-day estimates of: mortality, rehospitalization, and any one of 23 adverse events.

The primary outcome of 30-day mortality was defined within the manuscript text

The secondary outcome of rehospitalization was defined based on the data fields available and clinical expertise. Rehospitalization was defined by the following criteria:

- If a patient was discharged to home and experienced a subsequent inpatient or SNF admission within the time period of interest, the admission was classified as a rehospitalization.
- If a patient was discharged to a SNF, hospice, or another "long term care facility", and experienced a subsequent inpatient admission within the time period of interest, that admission was classified as a rehospitalization.
- If a patient was transferred to another inpatient facility, then this set of rules were applied to that subsequent inpatient admission (otherwise, the admission was not classified as a rehospitalization).

eFigure 1 shows a flow chart view of the logic applied in this set of rules.

eFigure 1– flowchart of the logic to classify the unplanned admission outcome

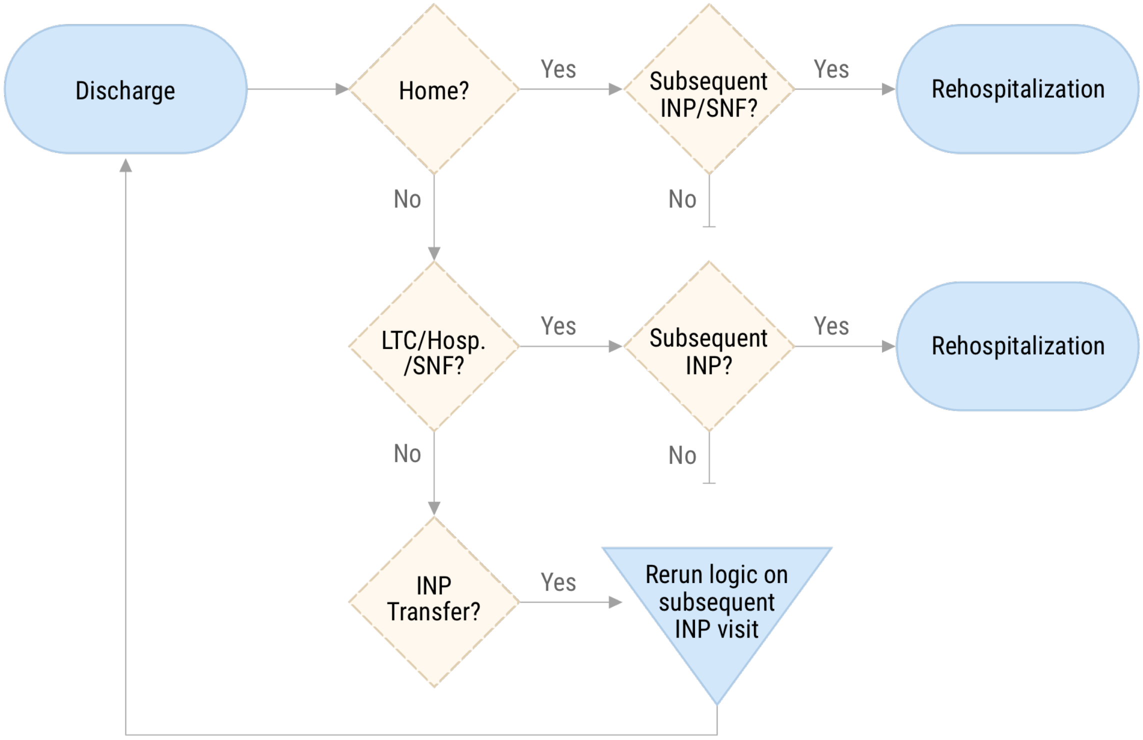

eFigure 1 legend: Starting in the upper left hand corner, a given beneficiary is discharged from an inpatient hospitalization to a given destination. After following the flow diagram, based on the above logic, the beneficiary was defined as either having an inpatient rehospitalization or not.

The secondary outcomes of adverse events were defined by data visualization in combination with clinical expertise as described below.

To determine which specific adverse events to focus on, we calculated the frequency of occurrence of each Hierarchical Condition Category (HCC) in the billing codes both before and after the index admission across all the patients. eFigure 2 shows a ranking of those HCC categories by change in the frequency of occurrence during and after the index admission as compared to frequency of occurrence prior to the index admission. An adverse event is an event that is more likely to develop during or after the index admission and thus would be more likely to be found towards the left-hand side of eFigure 2. This approach to adverse event classification was judged to be clinically sound, as it can be seen that the adverse events towards the left-hand side of eFigure 2 are more likely to be identified as "acute" disease complications associated with hospitalization opposed to chronic illnesses or diseases which are more often found to the right-hand side of eFigure 2. Therefore we focused our attention on adverse events listed towards the left-hand side of eFigure 2.

eFigure 2 – adverse events ranked by the change in occurrence frequency during and after the index admission as compared to prior to the index admission

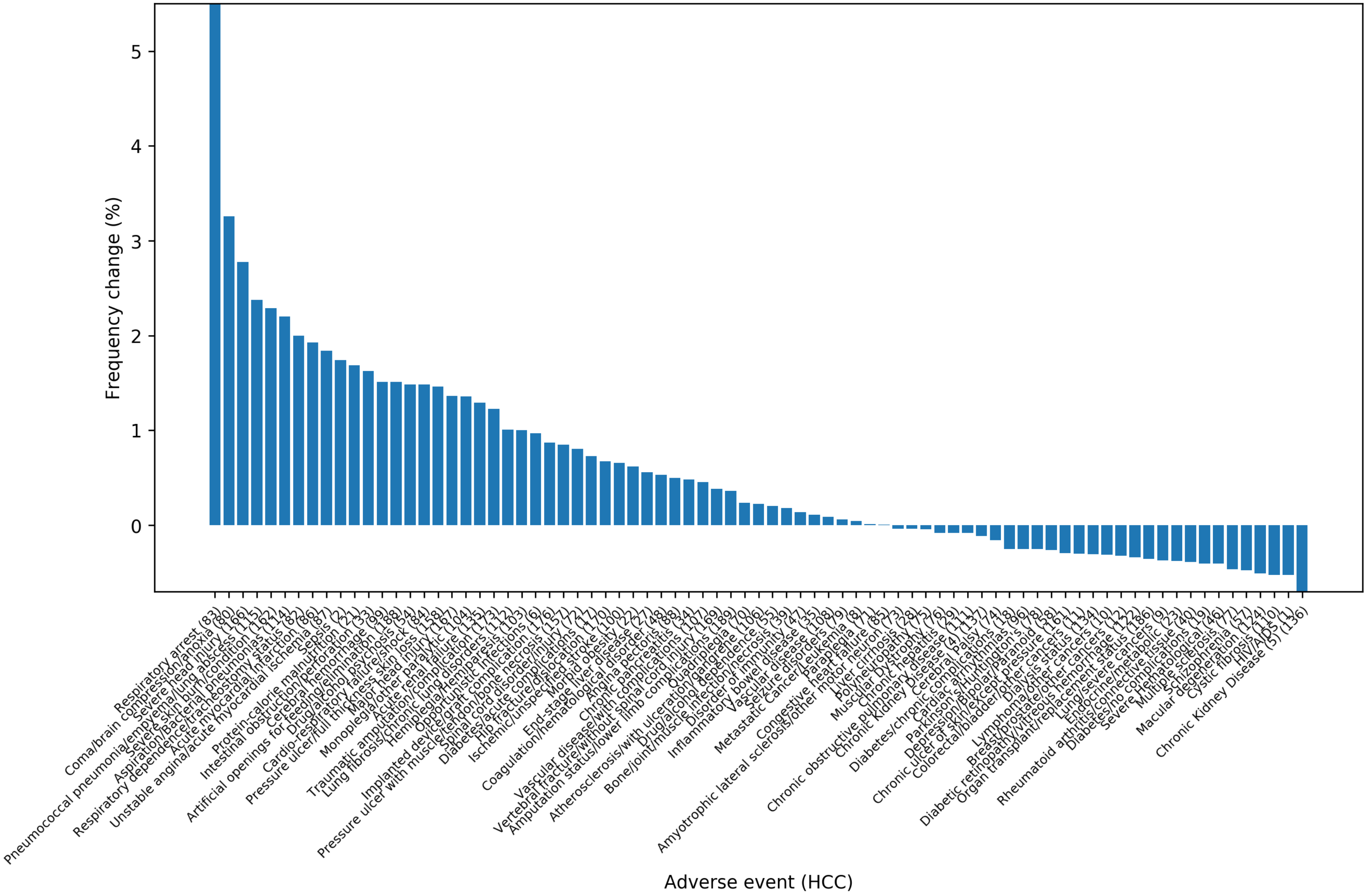

eFigure 2 legend: The categorized HCCs are plotted on the x-axis by frequency of occurrence on the y-axis – from most frequent (positive frequency change compared to during/after the index admission) to the least frequent (negative frequency change compared to during/after the index admission). Adverse events (i.e. acute conditions) appear on the left side – occurring with increased frequency during or after the index admission. Stable conditions (i.e. chronic conditions) appear more on the right side – occurring with decreased frequency during or after the index admission.

To further simplify the list of adverse events to predict, we grouped some of these HCC categories into buckets based on clinical expertise as a result of clinical similarity. See eTable 5 for full details.

eTable 5 presents a comprehensive list of the model outcomes, with adverse events bucketed for clinical similarity, for patients admitted between the dates of 01/01/2009 – 01/01/2011.

eTable 5

Target Events	Base Frequency
Mortality	8.7%
Unplanned admission<br>(inpatient or SNF)	9.1%
Respiratory arrest (HCC 83)	0.5%
Coma / brain compression/anoxia (HCC 80)	0.8%
Head trauma / brain bleed:<br>`Severe head injury (HCC 166)<br>`Cerebral hem. (HCC 99)<br>`Major head injury (HCC 167)	0.9%
Lung injury / pneumonia<br>`Pneumococcal pneumonia/empyema/lung abscess (HCC 115)<br>`Aspiration / bacterial pneumonias (HCC 114)	2.7%
Severe infection: skin / muscle / tendon / bone:<br>`Severe skin burn / condition (HCC162)<br>`Pressure ulcer / full thickness skin loss (HCC 154)<br>`Pressure ulcer muscle / tendon / bone necrosis (HCC 157)<br>`Bone / joint / muscle infection / necrosis (HCC 39)<br>`Atheromatous infection / ulceration / gangrene (HCC 106)	0.8%
Respiratory dependence / tracheostomy status (HCC 82)	0.9%
Myocardial ischemia / infarction:<br>`Acute myocardial infarction (HCC 86)<br>`Unstable angina / acute myocardial ischemia (HCC 87)	3.0%
Protein-calorie malnutrition (HCC 21)	2.2%
Sepsis (HCC 2)	3.5%
Intestinal obstruction / perforation (HCC 33)	2.4%
Drug / alcohol psychosis (HCC 54)	0.7%
Cardio-respiratory failure / shock (HCC 84)	6.0%
Artificial openings for feeding / elimination (HCC 188)	1.6%
Acute renal failure (HCC 135)	3.9%
Monoplegia / other paralytic (HCC 104)	0.3%
Limb amputation:<br>`Traumatic amputation / complication (HCC 173)<br>`Amputation status / lower limb complication (HCC 189)	0.7%
Opportunistic infections (HCC 6)	0.3%
Hemiplegia / hemiparesis (HCC 103)	1.5%
Implanted device / graft complication (HCC 176)	1.3%

Spinal cord injury / quadriplegia:<br>`Spinal cord dysregulation / injury (HCC 72)<br>`Quadriplegia (HCC 70)	0.8%
Hip fracture / dislocation (HCC 170)	0.6%
Diabetes / acute complication (HCC 17)	0.3%
Ischemic / unspecified stroke (HCC 100)	3.3%
