# Supplementary material for: Application of machine learning approaches to administrative claims data to predict clinical outcomes in medical and surgical patient populations
Source: PLoS One. 2021 Jun 3;16(6):e0252585. doi: 10.1371/journal.pone.0252585 (PMC8174683; doi:10.1371/journal.pone.0252585)
Supplement: S5 File — (PDF) [file pone.0252585.s005.pdf]

Supplement 5: Step 5 of Model Development – Evaluation of Model Strengths & Weaknesses

S5 Text: Step 5: Model Development and Evaluation of Strengths/Weaknesses

Step 5.a: Data preparation and Variable Weighting

All model development and evaluation work was performed using Python v3.7.6.(1) Notable open-source Python packages used were: NumPy(2), SciPy(3), Pandas,(4) SciKit-Learn,(5-7) and XGBoost.(8)

For the numerical variables in the raw input described in Step 4 above, missing values were imputed using the variable's median for the data set, and then each variable was independently scaled to have zero mean and unit standard variance.

The SciKit-Learn framework a-priori assumes all model inputs are numerical variables. Therefore, to prepare the raw input data for model development, all categorical variables needed to be transformed into numerical variables.

For "single category variables" in the raw input (that is, categorical variables that could only take on one category at a time for a single observation such as patient sex, race, or index admission DRG code) variables were transformed into numerical variables using one-hot encoding – that is, the encoded variable value is 1 for the column corresponding to the category, and 0 otherwise.

For "multiple category variables" in the raw input (that is, variables that contain a list of categories for a single observation such as the patient's history of diagnoses and procedure codes) variables were transformed into numerical variables using weighted multiple-hot encoding, with more frequent and more recent codes being given a higher value than less frequent and less recent codes. Specifically, the numerical value assigned for a given procedure or diagnosis code for an observation is determined by $\sum_i {d_i}^{\alpha}$ where i is the ith occurrence of that particular code in the patient's history, d_i is the number of days prior to the index admission that this occurrence of the code appeared, and α is a "decay rate" hyperparameter that can be adjusted to preferentially weight more recent code occurrences. After encoding, these multiple category variables were scaled to have unit difference between the 25th and 75th percentile range in order to prevent outliers, that is, those patients with a history of very many diagnoses and procedure codes, from unduly influencing the model fitting process. Alternative encoding approaches were also tried, such as creating a count variable for the total number of times a code was seen as well as a count variable for the number of times it was seen recently, but these alternative approaches didn't result in substantial model prediction differences. Different decay rate hyperparameters were explored but didn't result in substantial model prediction differences, so a constant decay rate of $\alpha = -0.2$ was used throughout in what follows.

eTable 6 provides a summary of how each variable was transformed and the resulting number of variables generated.

eTable 6

Variable	Type	Transformations	Number of resulting variables
Age, prior length of stay, and county census covariates: median income, % unemployed, % below poverty, household size, % married, % high school, % bachelor, % car commute to work	numerical	median imputer, standard scaler	10
Sex	categorical	one-hot encoding	2
Race	categorical	one-hot encoding	7
Admission source code	categorical	one-hot encoding	12
End-stage renal indicator code	categorical	one-hot encoding	2
Medicare status code	categorical	one-hot encoding	5
DRG code	categorical	one-hot encoding	26
Principal diagnosis code	categorical	one-hot encoding	79
Admitting diagnosis code	categorical	one-hot encoding	79
Prior procedure codes seen	categorical list	multiple-hot encoding, robust scaler	244
Prior diagnoses codes seen	categorical list	multiple-hot encoding, robust scaler	79
Total number of variables: 545[a]			
[a] Note that the 30 day unplanned admission model is designed to be used at time of patient discharge, and so also used length of stay (numerical) and the patient destination (categorical) variables for a total of 550 variables.			

Step 5.b: Model fitting and evaluation

For maximum flexibility in using the models in data visualization presentations and interpreting their predictions, we choose to develop a separate model for each time-period and model output combination. That is, we developed individual models to estimate the likelihood of mortality in 30 days, 90 days, 180 days, 365 days, the likelihood of unplanned admission in 30 days, and the likelihood of each of the 23 individual adverse events in 30 days, 90 days, 180 days, 365 days, and etc., for a total of 97 independent risk models.

We employed best-practices for objectively assessing model performance and robustness while guarding against overfitting. Specifically, we used 75% of the data for training a candidate model and the remaining 25% of the data to test and verify the accuracy and robustness of the model.

Predicting the model outputs described in Step 4 above can be interpreted as solving binary classification problems. For example, estimating the likelihood of a patient experiencing mortality, or the likelihood of an unplanned admission, or the likelihood of experiencing a particular adverse event within the given time window of the index admission. A broad suite of classification model types were applied to these problems and their prediction characteristics on the test data set were compared. We fit models of the following types: logistic regression, support vector machine, random forest, multi-layer perceptron neural net, and two variations of gradient boosted trees. In addition, we fit aggregate models comprised of combinations of two or more different types of models aggregated by consensus voting.

Model hyperparameters were tuned on the training data set using cross validation and final model predictions were made on the test data set. eTable 7 shows a summary of the area under the receiver operator curve (AUROC) performance metric for different model types fit to the same data set predicting the 30 day mortality outcome. As can be seen in eTable 7, the AUROC metric is quite close among all models, with XGB gradient boosted tree performing slightly better than the other models.

eTable 7

Model type	Model AUROC on test data set for 30 day mortality
Logistic regression (LR)	0.84
Support vector machine (SVM)	0.80
Random forest (RF)	0.86
Gradient boosted tree (GB)	0.86
Multi-layer perceptron (MLP) neural net	0.86
XGB gradient boosted tree (XGB)	0.88
Aggregate model (LR + GB + MLP)	0.86

In what follows, we chose to focus on two of these model types: Logistic regression (LR) because of its ease of clinical interpretability, and the XGB gradient boosted tree (XGB) because of its superior prediction accuracy. Each class of model will be fit independently for each outcome, and their results will be combined post-hoc in the patient visualization and interpretation. Please see eFigure 3 for more detailed metrics for the XGB and LR on the 30 day mortality test data set.

eFigure 3 – 30 day mortality LR and XGB model performance on the test data

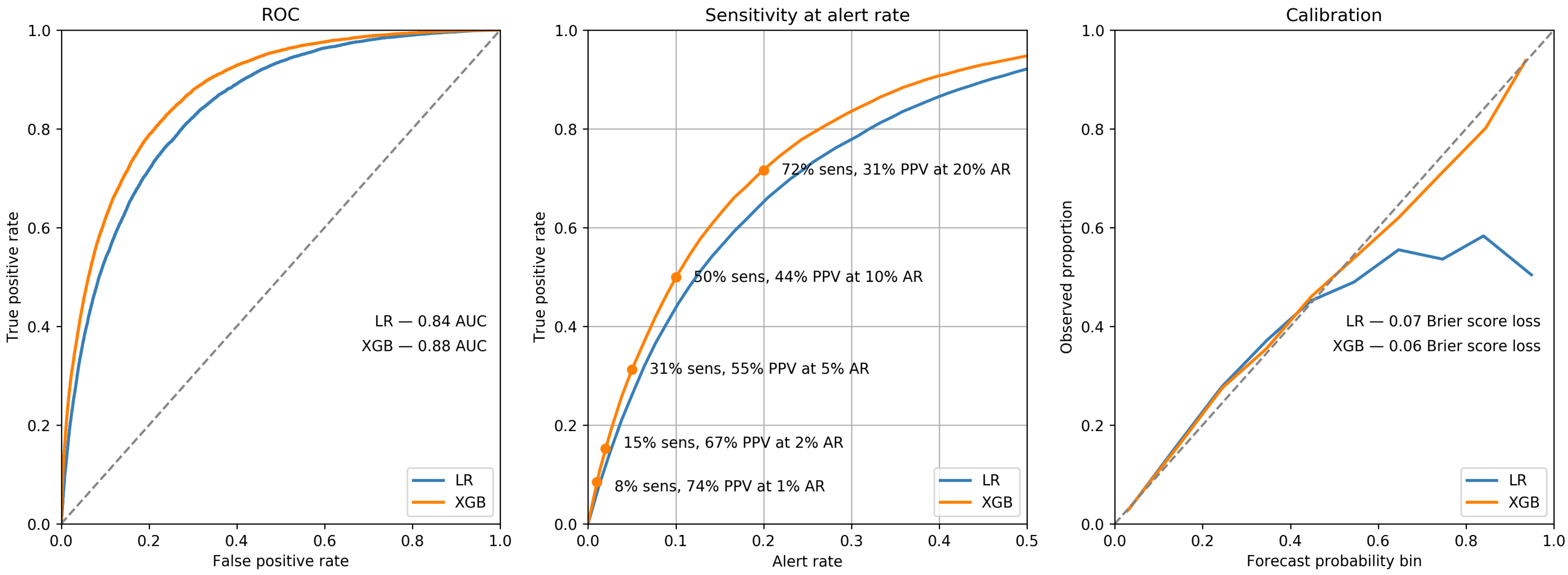

eFigure 3 Legend: The left most panel displays the full ROC curve. The middle panel of eFigure 3 displays the true positive rate vs the alert rate (e.g. 1% alert rate would capture the 1% at highest risk for suffering the adverse event); with selected alert rates (1%, 2%, 5%, 10%, and 20%) highlighted, along with details about the sensitivity and positive predictive value (precision) at those alert rates for the XGB (orange) and LR (blue) models. The right most panel of eFigure 3 displays calibration curves along with the Brier score loss – an indication of the overall calibration of the models.

eFigure 4 – 30-day mortality XGB model performance on the test data set

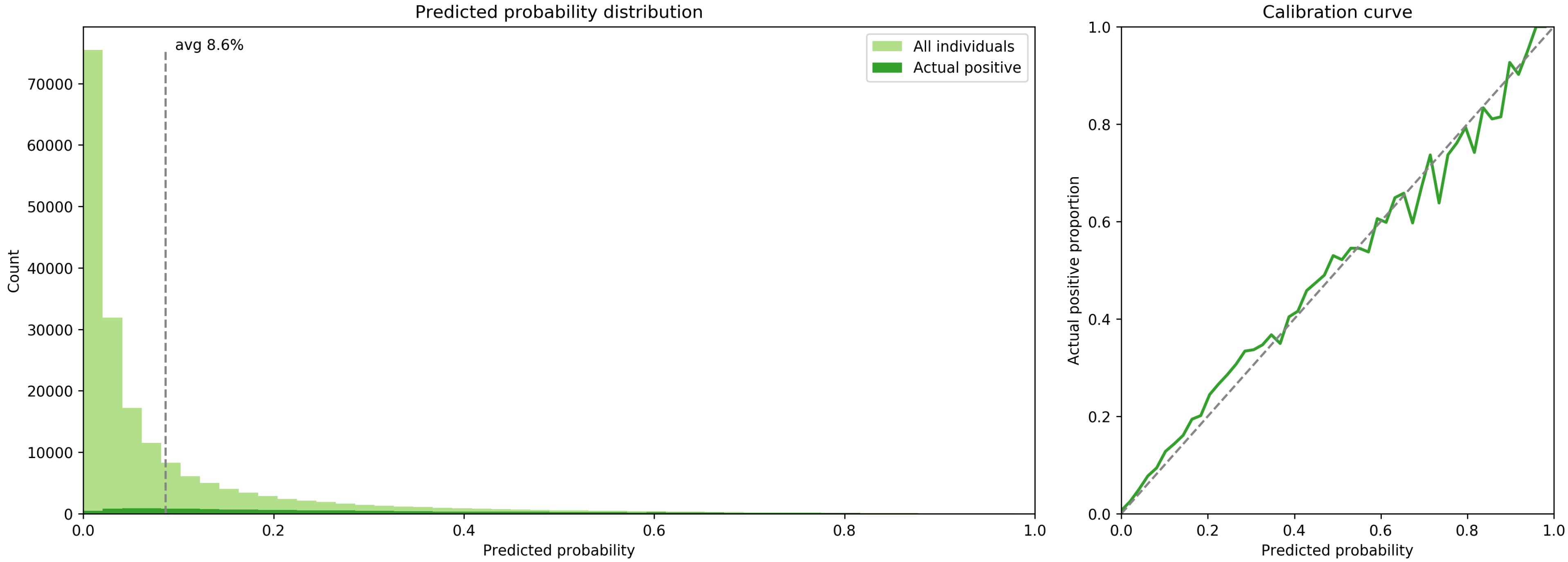

eFigure 4 Legend: The left panel shows that the XGB model produces a wide range of risk estimates for the test data set individuals, from 0.0 to 1.0. The right panel is a calibration curve and shows that each risk score bin accurately represents the true average mortality rate of those individuals in that bin. In other words the right panel shows that the predicted mortality curve (green line) follows the actual mortality curve (dashed line) closely.

eFigures 5 and 6 show the same model metrics for the 30 day unplanned admission model.

eFigure 5 – 30 day unplanned admission LR and XGB model performance on the test data set

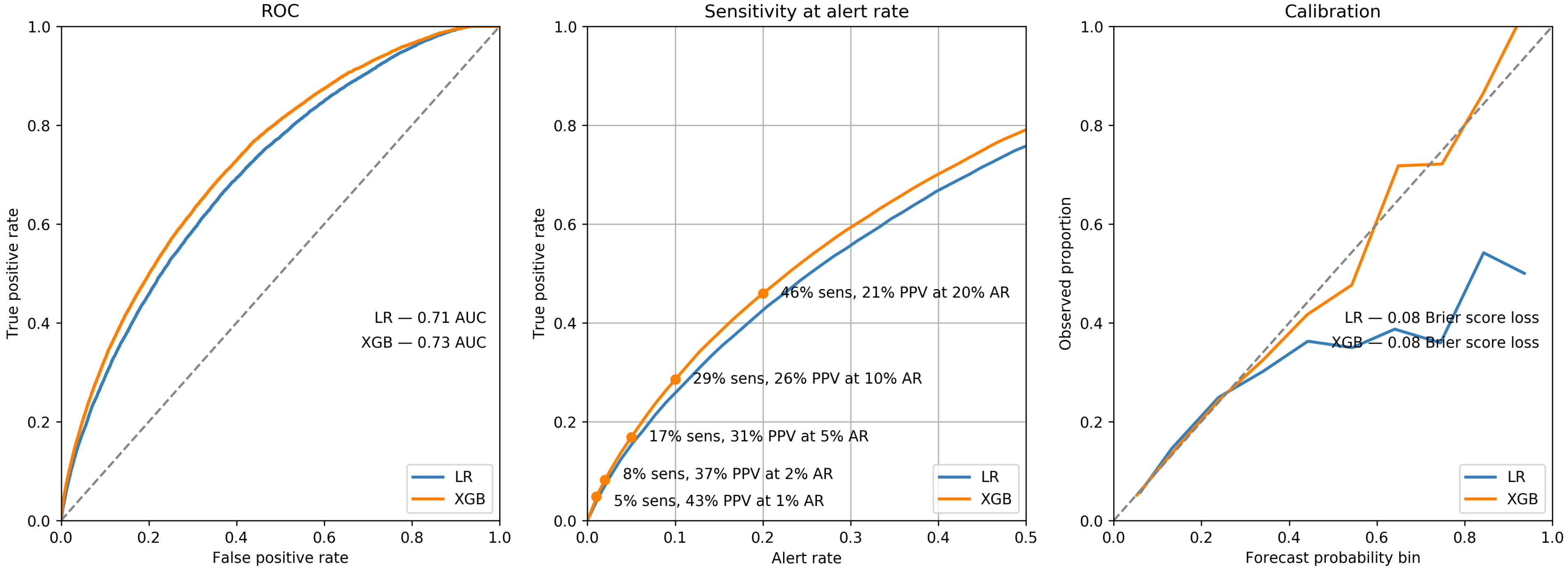

eFigure 5 Legend: The left most panel displays the full ROC curve. The middle panel displays the true positive rate vs the alert rate (e.g. 1% alert rate would capture the 1% at highest risk of rehospitalization); with selected alert rates (1%, 2%, 5%, 10%, and 20%) highlighted, along with details about the sensitivity and positive predictive value (precision) at those alert rates for the XGB (orange) and LR (blue) models. The right most panel displays calibration curves along with the Brier score loss – an indication of the overall calibration of the models.

eFigure 6 – 30 day unplanned admission XGB model performance on the test data set

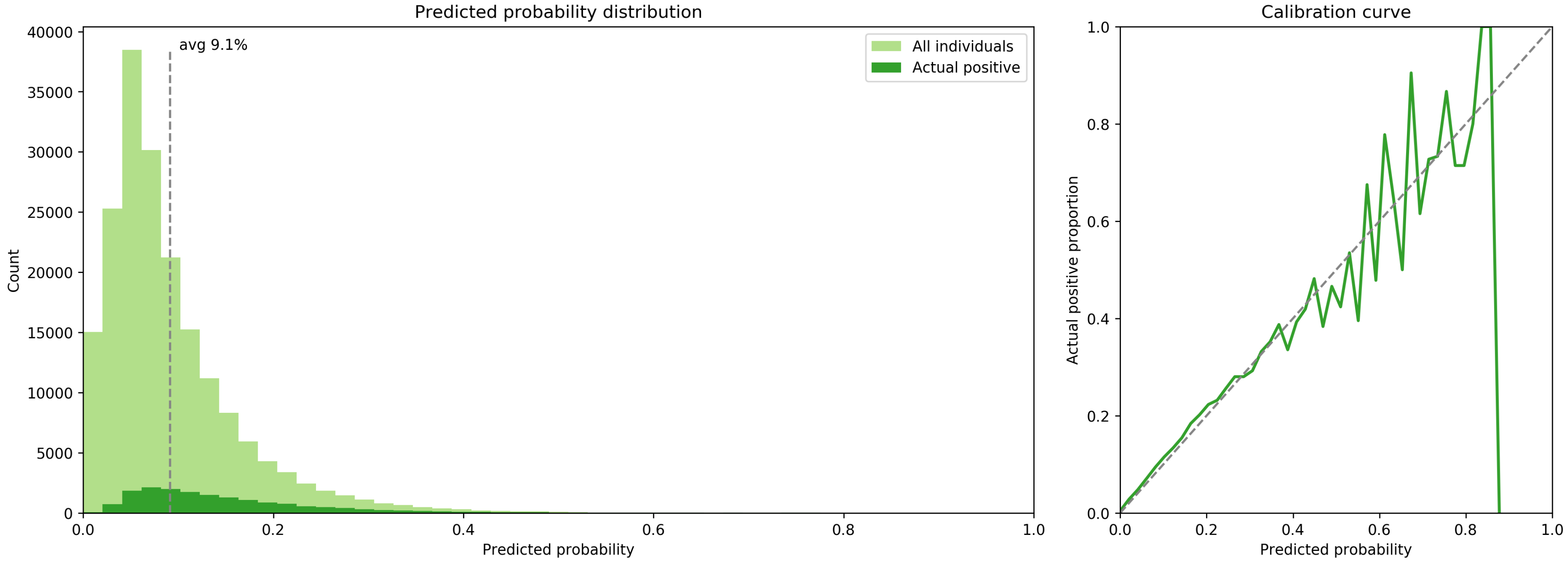

eFigure 6 Legend: The left panel shows that the XGB model produces a wide range of risk estimates for the test data set individuals, from 0.0 to 1.0. The right panel is a calibration curve and shows that each risk score bin accurately represents the true average rehospitalization risk of those individuals in that bin. As can be seen in the right panel, in contrast to the well-calibrated mortality model, as the risk of rehospitalization increases, the model becomes less calibrated – in other words the model follows the actual rate of rehospitalization at low rates, but “falls off” at higher rates of rehospitalization.

eFigures 7 and **8** show the same model metrics for the 30 day myocardial ischemia adverse event model. As can be seen in the figures, the XGB model has an AUROC metric of 0.89 on the test data set and is well calibrated overall. The other adverse events had similar model fitting results.

eFigure 7 – One example of the 23 adverse events – 30 day cardio-respiratory failure / shock LR and XGB model performance on the test data set.

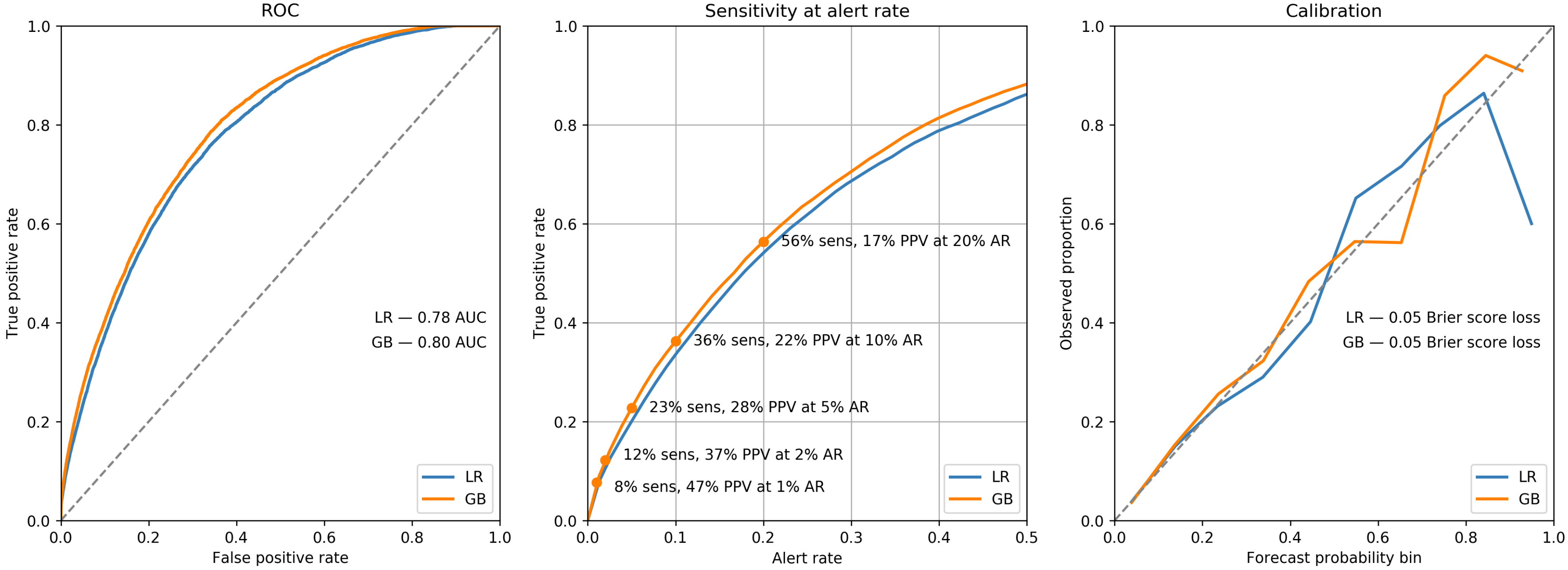

eFigure 7 Legend: The left most panel displays the full ROC curve. The middle panel displays the true positive rate vs the alert rate (e.g. 1% alert rate would capture the 1% at highest risk of rehospitalization); with selected alert rates (1%, 2%, 5%, 10%, and 20%) highlighted, along with details about the sensitivity and positive predictive value (precision) at those alert rates for the XGB (orange) and LR (blue) models. The right most panel displays calibration curves along with the Brier score loss – an indication of the overall calibration of the models.

eFigure 8 – One example of the 23 adverse events – 30 day cardio-respiratory failure / shock adverse event XGB model performance on the test data set.

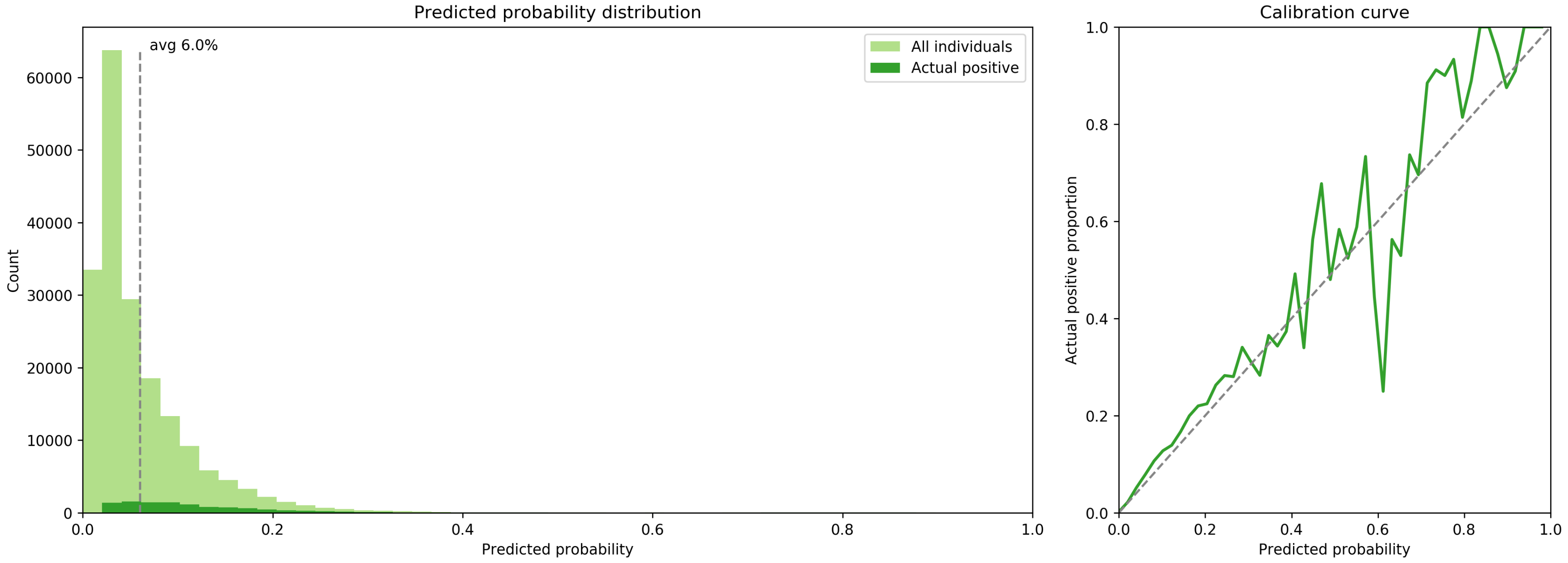

eFigure 8 Legend: The left panel shows that the XGB model produces a wide range of risk estimates for the test data set individuals, from 0.0 to 1.0. The right panel is a calibration curve and shows that each risk score bin accurately represents the true average rate of the given adverse event for individuals in that bin. In other words the right panel shows that the predicted risk of cardio-respiratory failure / shock curve (green line) follows the actual rate of cardio-respiratory failure / shock curve (dashed line) closely.

eFigure 9 – Adverse event model performance summary for all 22 adverse events of interest

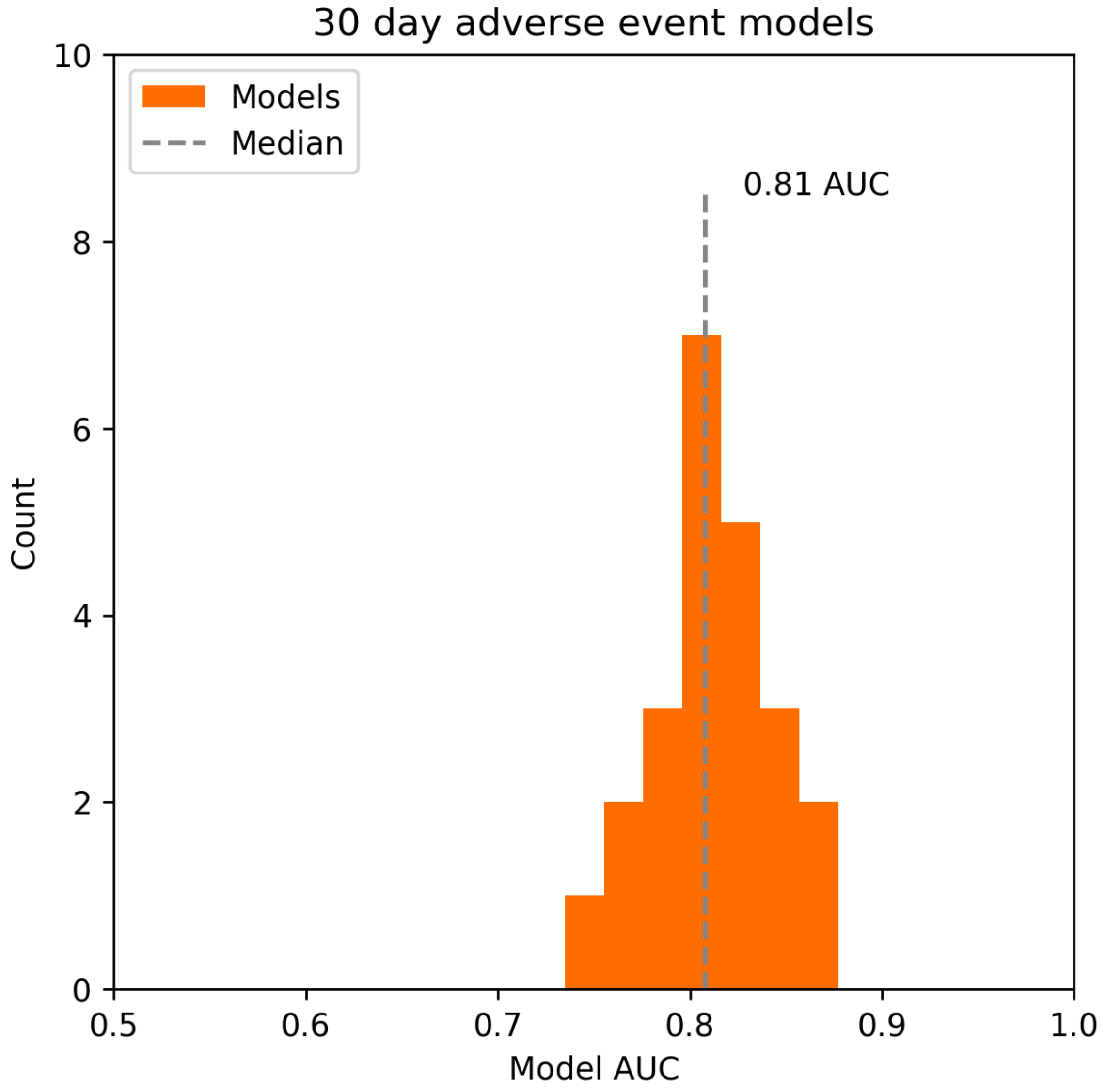

eFigure 9 Legend: median adverse event model performance for 30 days is 0.81 AUC with 0.75 the minimum AUC and 0.87 the maximum AUC across all the adverse event models. As might be expected, overall model performance degraded slightly as the time window was increased up to 1 year, because of the increased prediction time horizon.

S5 Supplement References

1. Language PP. Python v3.7.6 [Available from: https://www.python.org/.
2. Packages O-SP. NumPy [Available from: https://numpy.org/.
3. Packages O-SP. SciPy [Available from: https://www.scipy.org/.
4. Packages O-SPP. Pandas [Available from: https://pandas.pydata.org/.
5. Pedregosa F, Varoquaux G, Gramfort A, Michel V, Thirion B, Grisel O, et al. Scikit-learn: Machine learning in Python. Journal of machine learning research. 2011;12(Oct):2825-30.
6. Geron A. Hands-On Machine Learning with Scikit-Learn and TensorFlow. 1st Edition ed: O'Reilly Media, Inc., ; 2017.
7. Buitinck L, Louppe G, Blondel M, Pedregosa F, Mueller A, Grisel O, et al. API design for machine learning software: experiences from the scikit-learn project. arXiv preprint arXiv:13090238. 2013.
8. Package O-SPP. XGBoost [Available from: https://xgboost.readthedocs.io/
