# Supplementary material for: Application of machine learning approaches to administrative claims data to predict clinical outcomes in medical and surgical patient populations
Source: PLoS One. 2021 Jun 3;16(6):e0252585. doi: 10.1371/journal.pone.0252585 (PMC8174683; doi:10.1371/journal.pone.0252585)
Supplement: S6 File — (PDF) [file pone.0252585.s006.pdf]

Supplement 6: Model Performance on Independent, Test Dataset

Model Performance on Independent, Test Dataset

The ML risk model was retrained using all the original data and tested on an independent, test dataset as described in the manuscript. Model performance was assessed using the same parameters as the original model evaluation – AUROC and Brier Score and results are presented in eTable 8.

eTable 8 – Results of prediction model on independent test dataset

Target Events	Base Frequency	AUROC	95% CI for AUROC	Brier Score
Mortality	8.165%	0.875	[0.873-0.877]	0.058
Unplanned admission (inpatient or SNF)	4.587%	0.718	[0.714-0.721]	0.053
Acute renal failure (HCC 135)	2.592%	0.812	[0.809-0.816]	0.024
Cardio-respiratory failure / shock (HCC 84)	4.460%	0.823	[0.820-0.826]	0.039
Intestinal obstruction / perforation (HCC 33)	1.833%	0.812	[0.807-0.818]	0.017
Ischemic / unspecified stroke (HCC 100)	2.293%	0.841	[0.837-0.846]	0.020
Lung injury / pneumonia (HCCs 115, 114)	1.980%	0.813	[0.808-0.818]	0.018
Myocardial ischemia / infarction: (HCCs 86, 87)	1.980%	0.811	[0.806-0.816]	0.018
Protein-calorie malnutrition (HCC 21)	1.482%	0.790	[0.785-0.796]	0.014
Sepsis (HCC 2)	2.658%	0.817	[0.812-0.821]	0.024
